# Chronic Toxicity of Ferric Iron for North American Aquatic Organisms: Derivation of a Chronic Water Quality Criterion Using Single Species and Mesocosm Data

**DOI:** 10.1007/s00244-018-0505-2

**Published:** 2018-01-22

**Authors:** Pete Cadmus, Stephen F. Brinkman, Melynda K. May

**Affiliations:** 1 Aquatic Research Section, Colorado Parks and Wildlife, 317 West Prospect Rd., Fort Collins, CO 80526 USA; 2 Department of Fish, Wildlife and Conservation Biology, Colorado State University, Fort Collins, CO 80523 USA; 3 Water Resources Section, Colorado Parks and Wildlife, 6060 Broadway St., Denver, CO 80216 USA

## Abstract

Iron is a common pollutant in waters near coal and hard rock mine disturbances. The current 1000 µg/L total recoverable chronic criterion for iron (Fe) for protection of aquatic life in the United States was developed using very limited data in 1976 and has not been revised since. To develop a more scientifically based criterion, several chronic laboratory toxicity experiments (> 30 days) were conducted with ferric Fe at circumneutral pH on a taxonomically diverse group of organisms including brown trout (*Salmo trutta*), mountain whitefish (*Prosopium williamsoni*), boreal toad tadpoles (*Bufo boreas*), the oligochaete worm *Lumbriculus variegatus*, the mayfly *Hexagenia limbata*, and the planarian *Dugesia dorotocephala.* Results of these tests and those of previously published toxicity data were used to derive a Final Chronic Value (FCV) of 499 µg/L by using the US Environmental Protection Agency’s recommended methods based on single species toxicity tests. In addition to single species toxicity tests, ferric Fe toxicity experiments (10 days) were performed on mesocosms containing naturally colonized communities of benthic macroinvertebrates. Fourteen genera in the mesocosms occurred at sufficient densities to estimate an iron concentration resulting in 20% reduction in abundance (EC_20_). Three of these taxa had EC_20_s less than the FCV of 499 µg/L derived from single species tests: the mayfly *Epeorus* sp. (335 µg/L), the caddisfly *Micrasema* sp. (356 µg/L), and midge Tanytarsini (234 µg/L). When mesocosm results were included, the FCV was lowered to 251 µg/L. These findings support the suggestion that modernization of water quality criteria should include data generated from mesocosm experiments and other lines of evidence.

**Electronic supplementary material:**

The online version of this article (10.1007/s00244-018-0505-2) contains supplementary material, which is available to authorized users.

Iron is abundant in the earth’s crust and occurs naturally in the aquatic environment; however, concentrations can be elevated due to human activities. Mining activities that expose pyrite and other sulfidic minerals to air and water lead to oxidation and release of iron and sulfuric acid in a process known as acid mine drainage (AMD). An estimated 20,000 to 50,000 mines in the western United States produce AMD, which “seriously” affects 8000–15,000 km of streams (USDA 1993) and is considered the greatest water quality problem in the Rocky Mountain region (Mineral Policy Center 1997). In the eastern United States, acid drainage from coal mines affects more than 7000 km of streams (Kim et al. 1982). Despite the widespread and harmful effects of iron, fewer than half of U.S. states have adopted a numeric chronic iron standard to protect aquatic life, and several states have deleted iron standards. The current USEPA chronic iron criterion of 1000 µg/L (total recoverable) for protection of aquatic life was adopted in 1976 and is largely based on field observations of a single iron-polluted Colorado stream in which trout and other fishes were absent at iron concentrations > 1000 µg/L (USEPA 1976). A field study conducted in Kentucky supported the 1000 µg/L criterion (Birge et al. 1985). Nevertheless, the basis for this criterion is generally regarded to be insufficient (Thurston et al. 1979; Ohio EPA 1998). Development of a more scientifically rigorous iron criterion has been challenging because of its complex speciation, which is influenced by redox, dissolved oxygen, light, pH, and organic matter (Vuori 1995). In the aqueous environment, iron exists in two oxidation states: reduced ferrous ion (Fe II) and oxidized ferric ion (Fe III). In oxygenated waters, soluble ferrous ions (Fe II) oxidize to ferric ions (Fe III; Hem 1985). In circumneutral waters (pH > 6.5), ferric ions are insoluble and rapidly precipitate as hydroxides and oxyhydroxides (Hem 1985; Kimball et al. 2007). While iron speciation is indeed complex, ferric precipitates are the predominant form in waters capable of supporting aquatic life (i.e., oxygenated and circumneutral pH). Thus, ferric precipitates are the most relevant form of iron to consider for the development of a criterion for the protection of aquatic life.

U.S. water quality criteria are usually derived using the methodology outlined by Stephan et al. (1985). Briefly, a chronic criterion is intended to be protective of 95% of genera estimated from a dataset of toxicity values consisting of minimum of eight families that includes Salmonidae, another fish family in class Osteichthyes, a third family in Chordata, a planktonic crustacean, a benthic crustacean, an insect, a family in a phylum other than Arthropoda or Chordata, and finally a family in any order of insect or phylum not already represented. For our study, a literature review was conducted to identify chronic iron toxicity tests that met the following four criteria: (1) The species of test organisms used must exist in freshwater systems in North America; (2) The duration of the test was sufficiently long to detect sublethal effects (≥ 25 or ≥ 7 days for Daphnids); (3) Ferric iron was used as the toxicant, because precipitates are the overwhelmingly predominant form of iron in circumneutral oxygenated waters; and (4) Toxicity tests were conducted at pH between 6.5 and 9.0 to minimize confounding effects of pH on results (Radford 1997). Suitable tests existed for genera from Salmonidae (*Oncorhynchus, Salvelinus),* another fish from class Osteichthyes (*Pimephales*), a planktonic crustacean (*Daphnia*), a benthic crustacean (*Orconectes*), and an insect (*Chironomus*) (Table 1). To add to this existing dataset, we conducted chronic single species laboratory toxicity tests on a Chordate (*Bufo*), an insect (*Hexagenia*), a nonarthropod invertebrate (*Lumbriculus*), additional members of Salmonidae (*Salmo, Prosopium)*, and a family in another insect order or a phylum not otherwise represented (*Dugesia*). With these toxicity test results, iron toxicity data are available for a sufficiently diverse array of organisms to meet USEPA’s recommended methods to calculate a chronic final value (FCV) for total Fe.

**Table 1:** Chronic values from experiments included in calculation of species mean chronic values (SMCV) and genus mean chronic values (GMCV)

Rank	Scientific name	Common name	Chronic value	SMCV	GMCV	References
			(µg/L)	(µg/L)	(µg/L)	
12	*Dugesia dorotocephala*	Planarian	40,134	40,134	40,134	This study
11	*Orconectes limosus*	Crayfish	22,000	22,000	22,000	Boutet and Chasemartin (1973)
10	*Chironomus riparius*	Midge	19,818	19,811	19,811	Radford (1997)
9	*Salvelinus fontinalis*	Brook trout	9237	9237	9237	Sykora et al. (1975)
8	*Hexagenia limbata*	Mayfly	7863	7863	7863	This study
7	*Salmo trutta*	Brown trout	5146	5146	5146	This study
6	*Oncorhynchus kisutch*	Coho salmon	4870	3605	3467	Smith and Sykora (1976)
	*Oncorhynchus kisutch*	Coho salmon	3300			Brenner and Cooper (1978)
	*Oncorhynchus kisutch*	Coho salmon	2915			Updegraff and Sykora (1976)
	*Oncorhynchus mykiss*	Rainbow trout	1483	3335		Goettl and Davies (1977)
	*Oncorhynchus mykiss*	Rainbow trout	7500			Steffens et al. (1993)
5	*Bufo boreas*	Boreal toad (tadpole)	3145	3145	3145	This study
4	*Daphnia magna*	Cladoceran	4380	4380	2048	Biesinger and Christensen (1972)
	*Daphnia pulex*	Cladoceran	958	958		Birge et al. (1985)
3	*Prosopium williamsoni*	Mountain whitefish	1318	1318	1318	This study
2	*Lumbriculus variegatus*	Worm	870	870	870	This study
1	*Pimephales promelas*	Fathead minnow	910	688	688	Birge et al. (1985)
	*Pimephales promelas*	Fathead minnow	520			Smith et al. (1973)

Fe concentrations in µg/L total or total recoverable Fe. Excludes mesocosm experiments

Traditionally, results from single species toxicity tests are used exclusively to derive U.S. water quality criteria. While single species tests offer a greater degree of control and evidence for causation, they cannot evaluate interspecific interactions such as increased susceptibility to predation and ecologically relevant endpoints such as drift, which mesocosms are able to provide. A recent paper (Buchwalter et al. 2017) suggested that ecologically relevant lines of evidence be used in the creation of water quality criteria. Specifically, the authors recommended the inclusion of mesocosm data in criteria development and the immediate use of mesocosm studies to test the hypothesis that a criteria is protective. To test the hypothesis that our single-species derived FCV was protective, we conducted a 10 day mesocosm experiment using naturally colonized communities of benthic macroinvertebrates. The results from this experiment were compared to the Fe criterion calculated from the single species experiments. Lastly, effect concentration (EC_20_) values for species in the mesocosm experiments were incorporated into the species sensitivity distributions from single species tests to derive a FCV using both single species and mesocosm data.

## Methods

Single species test methods followed ASTM method E1241, *Standard Guide for Conducting Early Life*-*Stage Toxicity Tests with Fishes* (ASTM 1997) using ferric chloride as the toxicant. Dissolution of ferric chloride and the subsequent precipitation of ferric hydroxide release acidic protons according to the reaction:

$${\text{FeCl}}_{3} + 6{\text{H}}_{2} {\text{O}} \to {\text{Fe}}\left( {\text{OH}} \right)_{3} + 3{\text{H}}^{ + } + {\text{Cl}}^{ - }$$

As a result, adding a concentrated stock solution of ferric chloride to dilution water would lower pH and alkalinity and confound interpretation of toxicity results (Radford 1997). To prevent changes in pH and alkalinity among iron exposure levels, sodium hydroxide was added to the stock solution in a 3:1 stoichiometric ratio to neutralize the acid formed by the precipitation of ferric hydroxide. Stock solutions were > 6.5 pH before use. Measured alkalinity and pH were similar among the iron exposure levels for all studies. In the flow-through experiments, aeration of stock solutions, diluter compartments and exposure chambers were used to minimize settling of ferric precipitates.

### Brown Trout and Mountain Whitefish

Freshly fertilized eggs were collected from wild spawning adults. Brown trout (*Salmo trutta*) eggs and milt were collected as part of the annual Colorado Parks and Wildlife spawning operations (North Delaney Buttes Reservoir, Jackson County, CO). Mountain whitefish (*Prosopium williamsoni*) eggs and milt were collect from adults in spawning condition (Mad Creek, Routt County, CO). Eggs were stripped, fertilized, and water-hardened in the field and transported in coolers to the Colorado Parks and Wildlife (CPW) Aquatic Toxicology Laboratory (Fort Collins, CO). Upon arrival, eggs were treated with 1600 ppm of formalin for 15 min to control fungus (Piper et al. 1986).

A continuous-flow diluter (Benoit et al. 1982) constructed of Teflon, polyethylene, and polypropylene components delivered five exposure levels of iron hydroxide and an exposure control. Source water was dechlorinated municipal tap water (Fort Collins, CO). Target Fe concentrations were 0, 625, 1250, 2500, and 5000 μg/L total iron. To accommodate the additional aquaria needed to test two species simultaneously the number of exposure levels was reduced from five to four. Iron stock solution was prepared by dissolving ferric chloride hexahydrate (FeCl_3_·6H_2_O, Mallinckrodt analytical reagent grade) with sufficient NaOH (1:3 stoichiometry) to neutralize acidic conditions caused by precipitation of ferric hydroxide. The stock solution was pumped to the diluter with a peristaltic pump at a rate of 2 mL/min. A flow splitter equally allocated each iron concentration to each of six replicate 7.5 L glass aquaria at 30 mL/min. Aquaria, stock solutions, and diluter compartments were aerated to keep iron precipitates suspended in the water column. Exposure solutions were delivered via food-grade vinyl tubing to egg incubation cups constructed of 1000 μm nylon screen affixed to PVC pipe segments (53 mm I.D. X 75 mm) with aquarium-grade silicone adhesive. Each incubation cup was suspended in a 7-L glass aquarium with a standpipe that allowed the exposure solution to overflow into a temperature-controlled water bath. Thirty eggs were distributed to each incubation cup. Treatments were arranged so that each species was exposed to three replicates of each iron concentration. Treatments were randomized in complete blocks. Ambient fluorescent light (16 h:8 h photoperiod) provided illumination. Temperature of dilution water and water bath was initially 7 °C and then increased to 12 °C after hatch of whitefish (Online Resource Table S1). Mountain whitefish temperatures were assigned a low temperature during egg incubation and a higher temperature after hatch for three reasons: (1) Mountain whitefish eggs do not survive temperatures > 8 °C (Rajagopal 1979; Brinkman et al. 2013); (2) The egg stage was expected to be a sensitive life stage and lower incubation temperatures would extend exposure times; (3) Lower temperature during egg incubation is a more natural temperature regime for fall spawning species, such as mountain whitefish and brown trout.

Incubation cups were inspected daily for egg mortality and hatch. The first 12 brown trout eggs and first 15 whitefish eggs to hatch were carefully transferred from the incubation cup to the aquarium by using a glass tube and pipette pump. Remaining eggs in the incubation cups were monitored for hatching and removed once hatching was completed. Thus, hatching success for each species was based on 30 embryos in each incubation cup, whereas fry survival and growth were based on 12 and 15 fry transferred to the aquaria for brown trout and mountain whitefish, respectively. After absorption of the yolk-sac, brown trout fry were fed starter trout chow (Rangen soft-moist) five times per day with an automatic feeder at a rate of 5% body weight (BW)/day. Whitefish fry were fed < 24 h brine shrimp nauplii three times per day (1–2 times per day on weekends and holidays) at a rate of 5% BW/day. Tests were ended 30 days post-swimup and fry were terminally anesthetized with MS-222 and weighed (g). Total duration of exposure including embryo and larval stages was 79 days for brown trout and 78 days for mountain whitefish.

### Boreal Toad Tadpoles

Fertilized boreal toad eggs (*Bufo boreas*) were obtained from Trout Lake (Larimer County, Colorado). A continuous-flow diluter (described above) delivered five exposure levels of iron and an exposure control. Target concentrations of 0, 500, 1000, 2000, 4000, and 8000 μg/L total iron were delivered at a rate of 40 mL/min to 2.8 L polypropylene tanks at 20 °C using methods described above. Five tadpoles (c.a. stage 18; Gosner 1960) were carefully distributed into each tank (*n* = 4 replicate tanks) using a glass pipette. Tadpoles were fed (ad libitum) a mixture of Mazuri amphibian feed and powdered algae wafers (1:1) and a processed slurry of kale, mustard greens and squash. Tanks were cleaned to remove feces and excess food every 2 days. Tanks were monitored daily for mortality. After 35 days of exposure, tadpoles were terminally anesthetized with MS-222 and lengths (mm), weights (g) and developmental stage (Gosner 1960) measured for each tadpole.

### Lumbriculus

Toxicity trials of *Lumbriculus variegatus* were conducted using organisms from an onsite culture obtained from the USEPA laboratory in Duluth Minnesota. The onsite culture was maintained in a 39-L glass aquarium with washed coarse sand as a substrate and fed a slurry of trout starter feed. At the start of the experiment, 15 individuals were weighed and placed into each 2.8-L polypropylene exposure chamber. Each contained 150 mL of coarse washed sand and was maintained at 21 °C. Target concentrations of 0, 1000, 2000, 4000, and 8000 μg/L total Fe were delivered to five replicate treatment tanks per exposure level as described above. After 35 days of exposure, individuals in each tank were enumerated and weighed.

### Hexagenia

*Hexagenia limbata* nymphs were obtained from Aquatic Research Organisms (Hampton, NH). At the start of the experiment, 10 individuals were placed into each 2.7-L polypropylene exposure chamber maintained at 17 °C. Glass tubes 5-cm long and of varying inside diameters (4.9, 6, 7, 9 mm) were provided as artificial burrows (Fremling and Mauck 1980), which the nymphs readily adopted. Nymphs were fed 2.0 mL of a slurry consisting of 500 mL of yeast-trout chow-Cerophyl (YTC), 20 g of Tetramin fish food, and 5 g of wheatgrass. Target concentrations of 0, 500, 1000, 2000, 4000, and 8000 μg/L of total iron were delivered to four replicate 2.8-L treatment tanks per treatment level as described above. After 30 days of exposure, individuals in each tank were enumerated and weighed.

### Dugesia

Planarian worms (*Dugesia dorotocephala*) were field-collected near the outflow of the Colorado Parks and Wildlife Bellvue-Watson Rearing Unit (Larimer County, Colorado). Six individuals were randomly placed in each of 24 polystyrene petri dishes (145 × 20 mm) each containing 100 mL of exposure solution. Preliminary studies showed *Dugesia dorotocephala* to be tolerant. Target concentrations of 0, 2500, 5000, 10,000, 20,000, and 40,000 µg/L total iron were made using dechlorinated municipal tap water (Fort Collins, Colorado) and an iron stock solution of ferric chloride hexahydrate (FeCl_3_·6H_2_O, Mallinkrodt analytical reagent grade) with sufficient NaOH (1:3 stoichiometry) to neutralize acidic conditions caused by precipitation of ferric hydroxide. Petri dishes were renewed twice weekly with freshly prepared exposure solutions. Dishes were monitored daily for mortality and fissioning (asexual reproduction in which a single organism physically splits into two organisms). After 30 days, the test was terminated and all remaining planarians were enumerated and weighed.

### Aquatic Macroinvertebrate Community

In October 2010, effects of ferric Fe on communities of aquatic macroinvertebrates were measured by using stream mesocosms containing naturally colonized substrate (Clements 2004, Cadmus et al. 2018). Colonization trays (10 × 10 × 6 cm) filled with cobble were allowed to colonize for 32 days in the South Fork of the Michigan River, a stream originating from a wilderness area in the Routt National Forest (Colorado, USA). Trays were randomly assigned to coolers (4 trays in each) and were transferred to the 18 experimental streams at the Colorado State University Stream Research Laboratory (SRL) in Fort Collins, CO. Flow-through conditions were maintained at 1.0 L/min of untreated water from a reservoir fed by mountain streams (Horsetooth Reservoir, Fort Collins, CO). Peristaltic pumps delivered stock solution to experimental streams to create six ferric Fe treatments (*n* = 3; 0, 464, 944, 2425, 5238, and 14,073 µg/L) for 10 days. Stock solutions of Fe were neutralized with NaOH as described above. Reagents were mixed and vigorously aerated for 1 h. Before use, all stock solutions were tested for a circumneutral pH (6.5–7.5). After 10 days, all organisms retained in a 355-µm sieve were preserved in ethanol for identification to genus (tribe or subfamily for chironomids) and enumeration.

### Water Quality

For all single species experiments, unfiltered (total) samples for total iron were collected weekly from each exposure level in 60-mL, high-density polyethylene bottles (Nalgene). During the mesocosm experiment, total iron was sampled from each experimental unit every other day in 15-ml polypropylene centrifuge tubes (Falcon). Samples were immediately preserved with high-purity nitric acid (JT Baker) to pH < 2. Iron concentrations were measured using an Instrumentation Laboratory Video 22 (Allied Analytical Systems, Franklin, MA) atomic absorption spectrometer with air-acetylene flame and Smith–Hieftje background correction. The spectrometer was calibrated before each use, and the calibration curve was verified through analyses of external quality assurance samples (High Purity Standards, Charleston, SC). Sample splits and spikes were collected at each sampling event to verify analytical reproducibility and recovery.

Water quality (pH, temperature, dissolved oxygen, hardness, alkalinity, and conductivity) was assessed every other day from each experimental unit in the mesocosm trial. During single species trials water quality characteristics were measured weekly in all aquaria within a block. A different replicate was selected rotationally each week. Alkalinity and hardness were determined according to standard methods (APHA 1998). Dissolved oxygen and pH were measured with an electronic meter (Oakton Model 300 or YSI model 550a and 63) calibrated before each use. Conductivity was measured with an YSI model 35 or 63 conductance meter.

### Statistical Analyses

The maximum allowable toxicant concentration (MATC) was calculated as the geometric mean of the no-observed-effect concentration (NOEC) and lowest-observed-effect concentration (LOEC) of the most sensitive endpoint. These were determined with analysis of variance (ANOVA) using Toxstat version 3.5 software (West Inc. 1996, Cheyenne, WY). Hatching success and survival data were arcsine square root transformed prior to ANOVA. Normality and homogeneity of variances were tested using Chi square and Bartlett’s test, respectively. Treatment means were compared to the control using William’s one-tailed test (Williams 1971, 1972). The highest measured iron concentration not associated with a treatment effect (e.g., decreased survival, decreased body weight) was designated as the NOEC. The lowest measured iron concentration associated with a statistically significant treatment effect was designated as the LOEC. Use of NOEC and LOEC values has fallen out of favor with the scientific community (Warne and van Dam 2008; USEPA 1999). For this reason, we used the concentration predicted to cause a 20% reduction in survival or performance (EC_20_) when data were available. EC_20_s were calculated using USEPA’s Toxicity Relationship Analysis Program (TRAP version 1.30a; USEPA 2015a). Three parameter piecewise linear estimates of log transformed abundance data were used to calculate EC_20_ values of mesocosm results. Linear analysis of log transformed abundance data has been successfully employed in community mesocosm experiments exposing insect communities to toxicants (Clements et al. 2013). A threshold sigmoidal model was used to model single species results.

### Derivation of Final Chronic Value

USEPA’s ECOTOX database (USEPA 2015b) and science literature databases were used to identify iron toxicity tests that were of sufficient duration (≥ 25 days, Daphnids ≥ 7 days) to detect sublethal effects such as reduced growth or reproduction. Only studies that used the ferric iron and conducted at circumneutral pH (6.5–9.0) were included. If sufficient data were reported, regression analysis was used to calculate chronic values. USEPA’s Toxicity Relationship Analysis Program version 1.30a (TRAP), was used to determine EC_20_ for both single species laboratory tests and mesocosm tests. If insufficient partial effects were observed to produce a reliable estimate or if insufficient data were reported to run TRAP, MATCs, or other effect concentrations reported by the authors were used for chronic values. Chronic values of toxicity tests that met the screening requirements are reported in Table 1. Details of TRAP results and chronic values are reported in the Methods Narrative in the Online Resource. Species mean chronic values (SMCV) were calculated as the geometric mean of chronic values in the limited instances where multiple chronic values were available for the same species. Genus mean chronic values (GMCV) were calculated as the geometric mean of relevant SMCVs. GMCVs were ranked, and a final chronic value (FCV) was calculated by using methods described by Stephan et al. (1985).

To test the hypothesis that our FCV derived from single species tests is protective of natural benthic communities, EC_20_ values from the mesocosm study were compared to the FCV. Only 14 of the 40 taxa present occurred in sufficient abundance to calculate an EC_20_ or to demonstrate that there was no significant effect at our highest concentration. Given the short duration of the mesocosm experiment and the inclusion of two insect families in our FCV, we predicted that our FCV was sufficiently below the EC_20_ of any macroinvertebrate taxa in our mesocosm. Finally, a second FCV was calculated by adding the 14 genera from the mesocosm study to the species sensitivity distribution that included GMCVs from single species trials.

## Results

### Single Species Toxicity Tests

Details on water-quality measurements and toxicity endpoints can be found in the Online Resource Tables S1–S6. Single species toxicity tests were deemed acceptable based on ASTM criteria (1997). Hatch success of brown trout and mountain whitefish exceeded 80% in the control treatments. Posthatch survival was 91 and 84% in brown trout and mountain whitefish controls, respectively. Control survival was 100% for boreal toad tadpoles and 84% for *Hexagenia* nymphs. The number of individuals in control treatments increased by a factor of 7.5 and 1.4 for *Lumbriculus* and *Dugesia*, respectively. Measured dissolved oxygen concentrations were near saturation (mean 97%; range 82–105%) and biomass loading never exceeded 3.1 g/L or 0.15 g/L/24 h. Temperatures within each experiment were consistent among exposure levels. Alkalinity and pH measurements within each experiment were consistent throughout the duration of each test and also consistent among exposure concentrations demonstrating that ferric chloride was neutralized by the addition of sodium hydroxide.

**Fig. 1:**
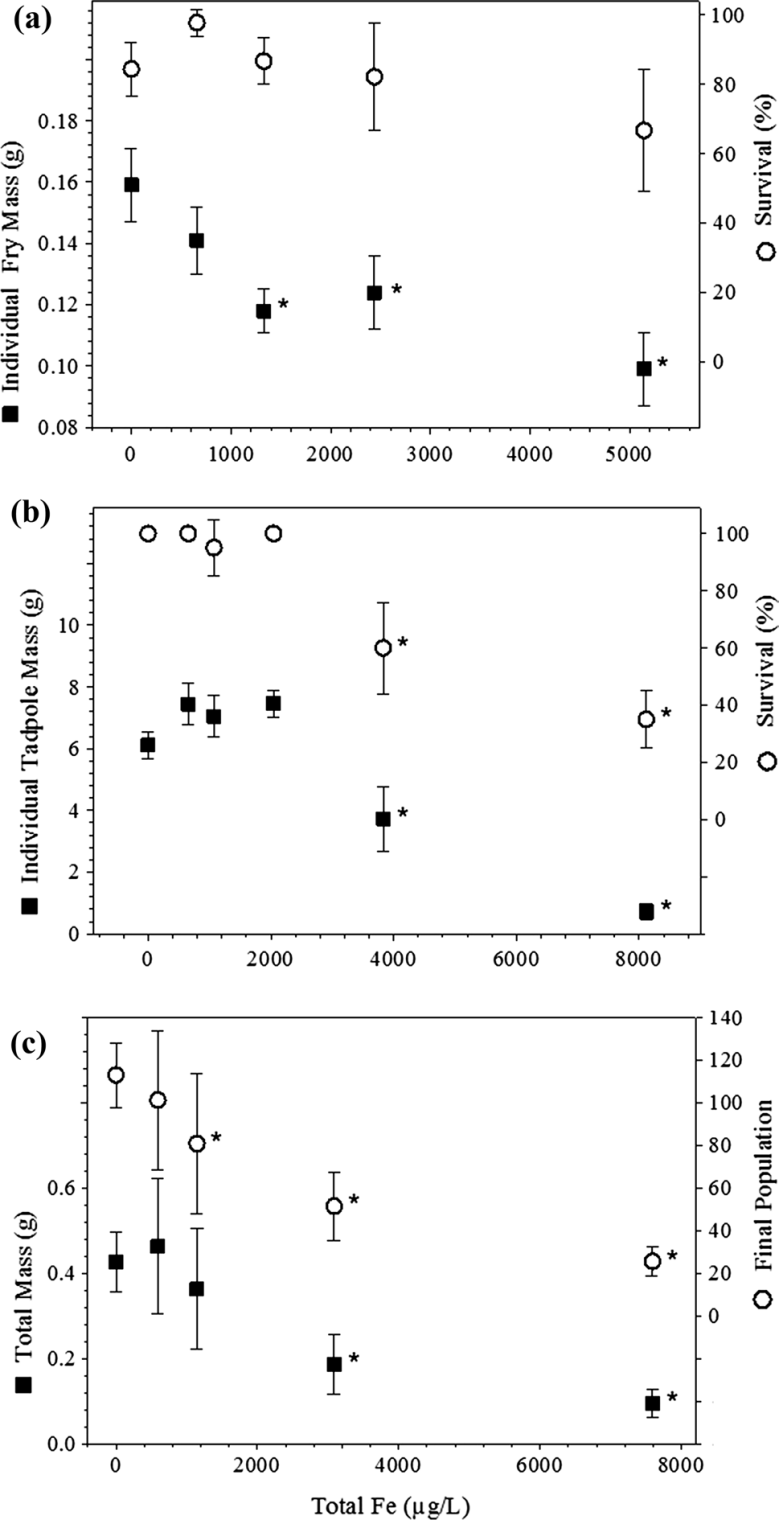
Response of organisms to total Fe. **a** Survival of mountain white fish was a less sensitive endpoint than growth measured in individual fry mass. **b** Reduced survival and mass of boreal toad tadpoles were observed at 3831 μg/L total iron. **c** Population size of *Lumbriculus* worms was reduced in the 1145 μg/L treatment levels when compared to controls. All experimental units started at 16 individuals. Asterisks denote treatment means significantly less than control (*p* < 0.05)

**Fig. 2:**
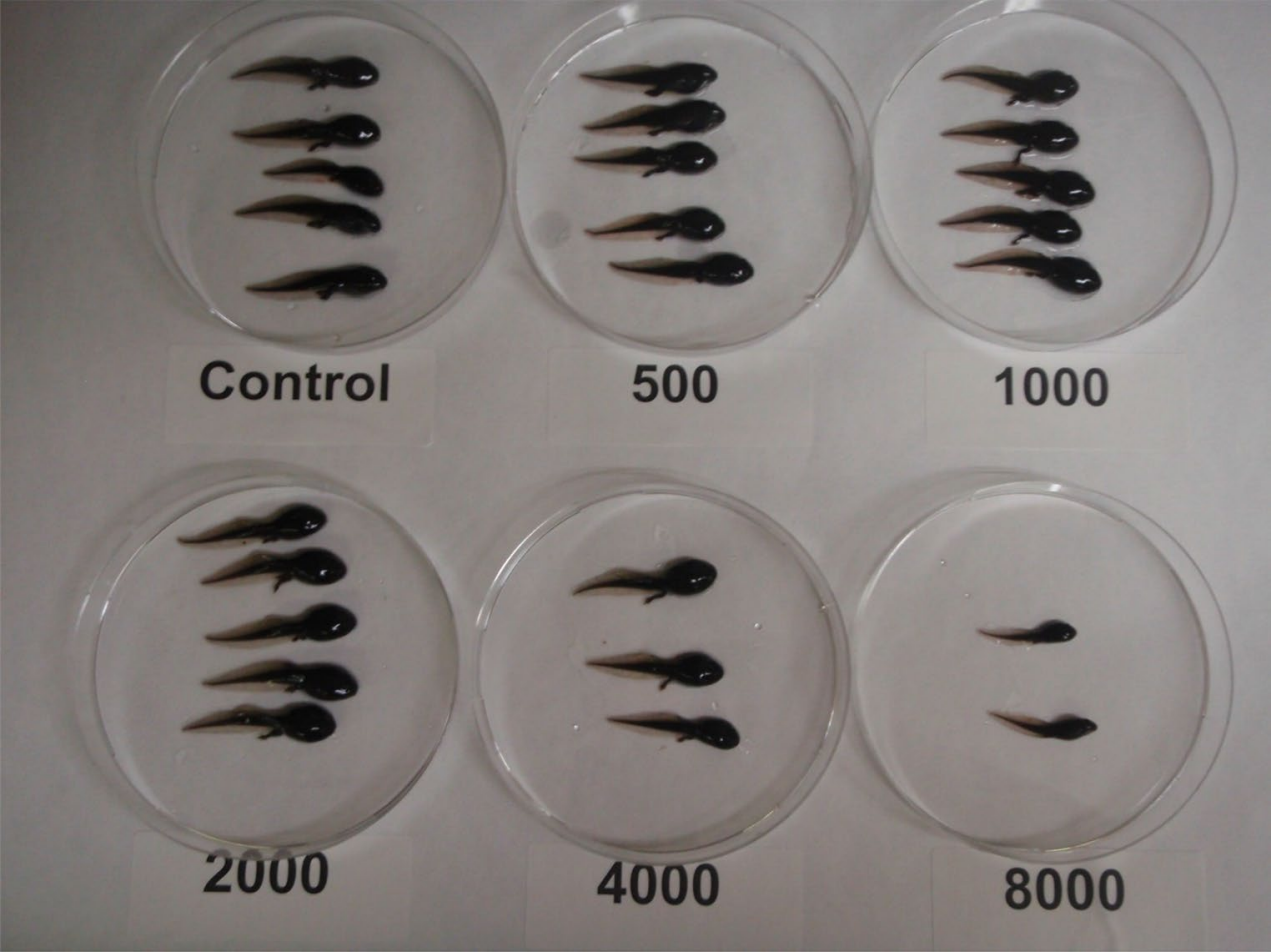
Boreal toad (*Bufo boreas*) tadpoles exposed to 8000, 4000, 2000, 1000, and 500 µg/L iron at termination of 35 days toxicity test. Tadpoles exposed to 8000 µg/L were significantly smaller and less developed than controls (*p* < 0.05)

Iron was not lethal to any of the organisms tested except for boreal toad tadpoles. Significant sublethal effects were detected including reduced growth for boreal toad tadpoles and mountain whitefish, reduced development for boreal toad tadpoles and reduced reproduction for *Lumbriculus* (Figs. 1, 2). EC_20_s were 1318 μg/L for mountain whitefish based on biomass, 3145 μg/L for boreal toad tadpoles based on biomass, and 870 μg/L for *Lumbriculus* based on number of organisms at the end of the test. No significant effects were detected for brown trout, *Hexagenia*, or *Dugesia* at exposure concentrations used in the tests.

### Final Chronic Value

Chronic toxicity data reported in the scientific literature were combined with results from this study (Table 1). Details on screening, acceptability and treatment of toxicity data from the literature are available in the Online Supplemental Narrative. Only 12 genera are represented, six of which are from this study. Although limited in taxonomic diversity and number of genera, the dataset met the eight-family minimum requirement needed to calculate a criterion (Stephan et al. 1985). Using the four most sensitive genera (*Pimephales, Lumbriculus, Prosopium,* and *Daphnia*) and 12 as the number of genera, a Final Chronic Value (FCV) of 499 µg/L total Fe was calculated.

### Mesocosm Toxicity Test

Physical and chemical assessments of experimental streams showed Fe concentrations near target levels and water quality similar to that of a high mountain stream (Online Resource Table S6). Despite the high-flow, turbulent environment produced in the experimental streams, we observed Fe precipitates clogging interstitial space and covering substrate and organisms. The Fe oxides appeared to indirectly affect benthos by reducing availability of benthic habitat, increasing turbidity, and reducing periphyton quality (Cadmus et al. 2018). Fourteen of the 40 aquatic insect taxa found in community mesocosms were sufficiently abundant (> 20 individuals in control streams) to calculate an EC_20_ value using TRAP (Table 2). EC_20_ values of the mayfly *Epeorus sp.* (335 µg/L), the caddisfly *Micrasema sp.* (356 µg/L), and the chironomid tribe Tanytarsini (234 µg/L) were below the FCV of 499 µg/L (Table 2). Additionally EC_20_ values of the chironomid subfamily Orthocladiinae (776 µg/L) were below the current national criterion of 1000 µg/L. If taxa from the mesocosm experiment are included, a FCV of 251 µg/L total Fe is calculated (Supplemental Material Table S9).

**Table 2:** EC_20_ values for genera (tribe for Chironomids) present in a 10 day mesocosm experiment exposing ferric iron to naturally colonized communities of benthic invertebrates

Genus (or tribe/subfamily)	EC_20_ (µg/L)
*Rhithrogena* sp.	> 14,073
*Ephemerella* sp.	> 14,073
*Sweltsa* sp.	> 14,073
*Brachycentrus* sp.	7558
*Baetis* sp.	4870
*Capnia* sp.	3697
*Cinygmula* sp.	1882
*Taenionema* sp.	1626
*Heterlimnius* sp.	1282
*Prostoia* sp.	1176
Orthocladiinae	776
*Micrasema* sp.	356
*Epeorus* sp.	335
Tanytarsini	234

## Discussion

Iron was not lethal in the single species toxicity tests except for boreal toad tadpoles. Instead, iron toxicity effects were sublethal, which included reduced growth for boreal toad tadpoles and mountain whitefish, reduced development for boreal toad tadpoles, and reduced reproduction for *Lumbriculus* (Figs. 1, 2). *Lumbriculus* are generally regarded as tolerant to dissolved metal exposure. However, iron precipitates that accumulated on the substrate may have interfered with feeding. This would be consistent with the notion that iron precipitates act as an indirect or physical stress on organisms and ecosystems rather than direct chemical toxicity. In neutral waters, Fe has been found to increase turbidity, reduce primary production, and reduce interstitial space in the benthic zones, which smothers invertebrates, periphyton, and eggs (USEPA 1976; Goettl and Davies 1977; DeNicola and Stapleton 2002; McKnight and Feder 1984; Vuori 1995; Linton et al. 2007; Hayer et al. 2013). Iron precipitates also physically clog and damage gills causing respiratory impairment (Peuranen et al. 1994; Dalzell and Macfarlane 1999).

The single-species FCV was calculated using EC_20_s as chronic values, in instances where EC_20_s could be reliably estimated. Otherwise, MATCs or other chronic toxicity values reported by authors were used. Chronic values based on regression analysis enable a uniform level of effect among different tests. In contrast, chronic values based on hypothesis testing to determine LOEC and NOEC are not based on magnitude of an effect and are sensitive to sample size, number of replicates, and variability of endpoints. Use of EC_20_s to derive FCVs was a risk-management decision made by the USEPA (1999), reflecting a compromise between a low level of effect, such as EC_10_, which is rarely significantly different from a control, and an EC_50_, which can be estimated with greatest precision but is clearly too large of an adverse effect for adequate protection. For the Fe chronic toxicity dataset, EC_20_s often were close to MATCs and nearly always between the NOECs and LOECs (Online Resource Table S6). Nevertheless, a FCV calculated using MATCs would have increased to 628 µg/L from the 499 µg/L calculated using EC_20_s.

**Fig. 3:**
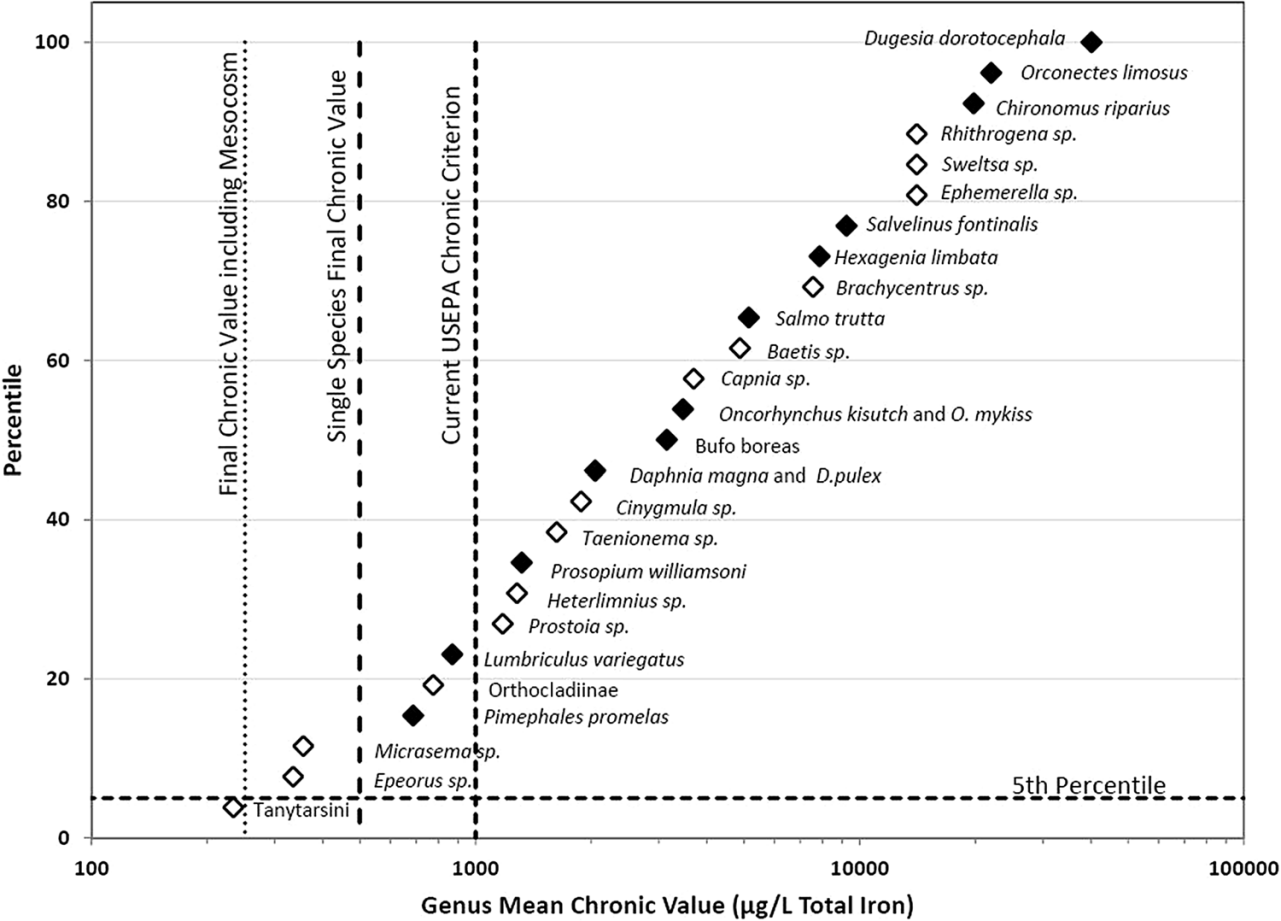
Sensitivity distribution of genera to iron. Filled diamond = chronic single species experiments. Diamond = 10 day mesocosm experiment

The current USEPA chronic Fe criterion for protection of aquatic life is 1000 µg/L total Fe, a value based principally on limited field observations which has not been updated since 1976. Using single species toxicity data in Table 1, a final chronic value of 499 µg/L total Fe was calculated by applying USEPA methodology (Stephan et al. 1985). We believe that this FCV is more rigorous and has a stronger scientific basis than the current criterion. This methodology uses chronic values of the four most sensitive genera to estimate a concentration that would protect 95% of taxa (Stephan et al. 1985). Interestingly, extrapolation of the trendline of the percentile versus genus mean chronic values of all genera to the 0.05 percentile yielded a concentration of 439 µg/L (Fig. 3), in good agreement with 499 µg/L derived using USEPA methodology.

Results from single species toxicity tests are currently the preferred data for deriving USEPA water-quality criteria. Such laboratory tests provide a high degree of control, standardization, and reproducibility. However, restricting water quality criteria to single-species data clearly has its limitations. Single-species tests lack environmental realism, rely on a limited number of easy to culture organisms, and do not consider interactions at higher levels of biological organization. Buchwalter et al. (2017) argued that water-quality criteria should incorporate more ecologically relevant data. One recommendation is to include results of mesocosm experiments. Indeed, results of the mesocosm experiment identified three taxa that would not be protected by the FCV calculated from single species tests. EC_20_ values of the mayfly *Epeorus sp.* (335 µg/L), the caddisfly *Micrasema* sp. (356 µg/L), and the chironomid tribe Tanytarsini (234 µg/L) were below the FCV of 499 µg/L derived using single species toxicity tests (Table 2). These findings highlight the limitations of single species based criterion values and strongly support inclusion of mesocosm test results for derivation of water quality criteria. Including mesocosm data would lower the FCV to 251 µg/L total Fe. The FCV with the mesocosm data included is supported by an assessment of field data that found iron as low as 210 µg/L may be necessary to protect sensitive insect species (Linton et al. 2007).

Inclusion of our benthic mesocosm results clearly adds environmental relevance and reduces the risk of calculating an underprotective FCV for total Fe. However, mesocosm experiments are not a panacea for improving water quality standards, particularly if they are poorly designed or are unrepresentative of natural biota. Improperly designed mesocosm experiments can overestimate safe pollution levels in the same ways as single species experiments. During this exercise we observed several qualities of mesocosm data that should be considered when deriving a standard with this approach. A single mesocosm experiment has potential to drastically influence a water quality standard. The derivation of water quality criteria described by Stephan et al. (1985) considers the four most sensitive species, whereas the addition of more tolerant species (Number of Genera) increases the FCV. In one single mesocosm experiment, we considered 40 macroinvertebrate taxa. Fourteen of these taxa were abundant enough to safely calculate an EC_20_. Our original FCV would have increased 250 or 148%, respectively, if the species considered were more tolerant than the fourth most sensitive genus (*Daphnia*) in the species sensitivity distribution. Standards are best informed by mesocosm experiments when those experiments represent the natural community and thus avoid consideration of an unnatural number of tolerant species or tolerant age classes. For this reason, we used naturally colonized communities from a pristine stream. Sampling disturbed sites or artificially building communities from tolerant species might have artificially inflated our FCV. Our experiment considered 40 species but only 12 were abundant enough to derive an EC_20_ value using TRAP. A larger sample size (more naturally colonized substrate) in each experimental unit would have allowed for inclusion of the more rare and sensitive species, better characterizing the entire community. In this experiment, we collected organisms retained in a 355-micron sieve. Use of a larger mesh size when sampling aquatic macroinvertebrates might underrepresent smaller, more sensitive, age classes. Additionally, community structure of benthos and organism size changes seasonally. To best characterize sensitivity of each taxa, mesocosm experiments might need to be repeated through the seasons. Improper design or methods can easily lead to underprotective FCV. However, we struggled to envision a situation in which similar mesocosm experiments could lead to an overprotective FCV when species composition of experiments is created using natural colonization from environmentally relevant locations.

The 10-day duration of our mesocosm experiment likely underrepresented the toxic effects of Fe that could be possible after 30 days or after a complete life cycle for these organisms. Shorter exposure durations typically increase the concentration at which a response is detectable. Mesocosm techniques using naturally colonized benthos have been conducted upwards of 30 days durations (Mebane et al. 2016). It is likely that increased exposure durations would have produced much lower FCV than we report.

Results of toxicity tests reported here suggest that the current USEPA chronic Fe criterion of 1000 µg/L is underprotective of sensitive aquatic life. Our FCV calculated from single-species toxicity tests suggests that the current criterion should be reduced by half to 499 µg/L. Mesocosm results and field data (Linton et al. 2007) suggest that sensitive species may require the Fe criterion to be reduced by half again to 251 µg/L. Some field studies observed that aquatic life appear unaffected at iron concentrations that exceed the water quality criterion of 1000 µg/L (Ohio EPA 1998; Loeffelman et al. 1985). Water-quality criteria are intended to protect 95% of species and, as such, may appear overly protective in circumstances where more tolerant organisms are present or in communities where sensitive species have been extirpated. Field studies that fail to detect adverse effects to aquatic life at concentrations above a criterion value should not necessarily be interpreted as demonstrating an overprotective criterion.

## Electronic supplementary material

Below is the link to the electronic supplementary material.

Supplementary material 1 (DOCX 66 kb)

## References

[CR100] APHA (1998) Standard methods for the examination of water and wastewater, 20th edn. American Public Health Association, American Water Works Association and Water Environmental Federation, Washington, DC

[CR1] ASTM (1997). Standard guide for conducting early life-stage toxicity tests with fishes. Standard E 1241-05.

[CR2] Benoit DA, Mattson VR, Olsen DC (1982). A continuous flow mini-diluter system for toxicity testing. Water Res.

[CR3] Biesinger KE, Christensen GM (1972). Effects of various metals on survival, growth, reproduction and metabolism of *Daphnia magna*. J Fish Res Board Can.

[CR4] Birge WJ, Black JA, Westerman, AG, Short TM, Taylor SB, Bruser DM, Wallingford ED (1985) Recommendations on numerical values for regulating iron and chloride concentrations for the purpose of protecting warm-water species of aquatic life in the commonwealth of Kentucky. Memorandum of Agreement No. 5429

[CR5] Boutet C, Chasemartin C (1973). Proprietes toxiques specifiques des sels metalliques chez Austropotamobius pallipes pallipes et Orconectes limosus. C R Seances Soc Biol Fil.

[CR6] Brenner FJ, Cooper WL (1978). Effect of suspended iron hydroxide on the hatchability and embryonic development of coho salmon. Ohio J Sci.

[CR7] Brinkman SF, Crockett HJ, Rogers KB (2013). Upper thermal tolerance of mountain whitefish eggs and fry. Trans Am Fish Soc.

[CR8] Buchwalter DB, Clements WH, Luoma SN (2017). Modernizing water quality criteria in the United States: a need to expand the definition of acceptable data. Environ Toxicol Chem.

[CR9] Cadmus P, Guasch H, Herdrich AT, Bonet B, Urrea G, Clements WH (2018). Structural and functional responses of periphyton and macroinvertebrate communities to ferric Fe, Cu and Zn in stream mesocosms. Environ Toxicol Chem.

[CR10] Clements WH (2004). Small-scale experiments support causal relationships between metal contamination and macroinvertebrate community responses. Ecol Appl.

[CR11] Clements WH, Cadmus P, Brinkman SF (2013). Responses of aquatic insects to Cu and Zn in stream microcosms: understanding differences between single species tests and field responses. Environ Sci Technol.

[CR12] Dalzell DJB, Macfarlane NAA (1999). The toxicity of iron to brown trout and effects on the gills: a comparison of two grades of iron sulphate. J Fish Biol.

[CR13] DeNicola DM, Stapleton MG (2002). Impact of acid mine drainage on benthic communities in streams: the relative roles of substratum vs. aqueous effects. Environ Pollut.

[CR14] Fremling CR, Mauck WL, Buikema AL, Cairns J (1980). Methods for using nymphs of burrowing mayflies (Ephemeroptera, *Hexagenia)* as toxicity test organisms. Aquatic invertebrate bioassays. ASTM STP 715.

[CR15] Goettl JP, Davies PH (1977). Study of the effects of metallic ions on fish and aquatic organisms. Water pollution studies, federal aid in fish and wildlife restoration project F-33-R12.

[CR16] Gosner KL (1960). A simplified table for staging anuran embryos and larvae with notes on identification. Herpetologica.

[CR17] Hayer CA, Holcomb BM, Chipps SR (2013). Associations between iron concentration and productivity in montane streams of the Black Hills, South Dakota. Prairie Nat.

[CR18] Hem JD (1985). Study and interpretation of the chemical characteristics of natural water. USGS. Water supply paper 2254.

[CR19] Kim AG, Heisey B, Kleinmann R, Duel M (1982) Acid mine drainage: control and abatement research. US Department of Interior, Bureau of Mines Information Circular 8905, pp 21–22

[CR20] Kimball BA, Walton-Day K, Runkel RL, Church SE, von Guerard P, Finger SE (2007). Quantification of metal loading by tracer injection and synoptic sampling 1996–2000. Integrated investigations of environmental effects of historical mining in the Animas River Watershed, San Juan County, Colorado.

[CR21] Linton TK, Pachego MA, McIntyre DO, Clement WH, Goodrich-Mahoney J (2007). Development of bioassessment-based benchmarks for iron. Environ Toxicol Chem.

[CR22] Loeffelman PH, Van Hassel JH, Arnold TE, Hendricks JC (1985) A new approach for regulating iron in water quality standards. In: Bahner RC (ed) Aquatic toxicology and hazard assessment, 8th symposium. ASTM STP 891. ASTM, Philadelphia, pp 137–152

[CR23] McKnight DM, Feder GL (1984). The ecological effect of acid conditions and precipitation of hydrous metal oxides in a Rocky Mountain stream. Hydrobiologia.

[CR24] Mebane CA, Schmidt TS, Balistrieri LS (2016). Larval aquatic insect responses to cadmium and zinc in experimental streams. Environ Toxicol Chem.

[CR25] Mineral Policy Center (1997). Golden dreams, poisoned streams.

[CR26] Ohio EPA (1998) Empirically derived guidelines for determining water quality standards and NPDES discharge limits for iron protective of aquatic life in Ohio rivers and streams. Technical Bulletin MAS/1998-9-1. Ohio EPA, Division of Surface Water, Columbus

[CR27] Peuranen S, Vuorinen PJ, Vuorinen M, Hollender A (1994). The effects of iron, humic acids and low pH on the gills and physiology of brown trout (*Salmo trutta*). Ann Zool Fenn.

[CR28] Piper RG, McElwain IB, McCraren JP, Fowler LG, Leonard JR (1986). Fish hatchery management.

[CR29] Radford NP (1997) Ecotoxicological impact of iron III sulphate dosing on chironomid cultures and profundal reservoir communities. Doctoral Thesis, University of Leicester

[CR30] Rajagopal PK (1979). The embryonic development and the thermal effects on the development of the Mountain Whitefish, *Prosopium williamsoni* (Girard). J Fish Biol.

[CR31] Smith EJ, Sykora JL (1976). Early developmental effects of lime-neutralized iron hydroxide suspensions on brook trout and coho salmon. Trans Am Fish Soc.

[CR32] Smith EJ, Sykora JL, Shapiro MA (1973). Effect of neutralized iron hydroxide suspensions on survival, growth and reproduction of the fathead minnow (*Pimephales promelas*). J Fish Res Board Can.

[CR33] Steffens W, Mattheis TH, Riedel M (1993). Field observations on the production of Rainbow Trout (*Oncorhynchus mykiss*) under high concentrations of water-borne iron. Aquat Sci.

[CR34] Stephan CE, Mount DI, Hansen DJ, Gentile JR, Chapman GA, Brungs WA (1985). Guidelines for deriving numerical standards for the protection of aquatic organisms and their uses. PB85-227049.

[CR35] Sykora JL, Smith EJ, Synak M, Shapiro MA (1975). Some observations on spawning brook trout (*Salvalinus fontinalis* Mitchell) in lime neutralized iron hydroxide suspensions. Water Res.

[CR36] Thurston RV, Russo RC, Fetterolf CM, Edsall TA, Barber YM (1979). A review of the EPA red book: quality criteria for water.

[CR37] Updegraff KF, Sykora JL (1976). Avoidance of lime-neutralized iron hydroxide solutions by coho salmon in the laboratory. Environ Sci Technol.

[CR38] USDA Forest Service (1993). Acid mine drainage from mines on the national forests: a management challenge.

[CR39] USEPA (1976). Quality criteria for water. PB263-943.

[CR40] USEPA (1999). 1999 update of aquatic life ambient water quality criteria for ammonia. EPA 822-R-99-014.

[CR41] USEPA (2015). Toxicity relationship analysis program (TRAP version 1.30a).

[CR42] USEPA (2015b) ECOTOX User guide: ECOTOXicology knowledgebase system. Version 4.0. https://cfpub.epa.gov/ecotox/blackbox/help/userhelp4.pdf. Accessed 28 April 2017

[CR43] Vuori KM (1995). Direct and indirect effects of iron on river ecosystems. Ann Zool Fenn.

[CR44] Warne MSJ, van Dam R (2008). NOEC and LOEC data should no longer be generated or used. Autralasian J Ecotoxicol.

[CR45] West Inc (1996). Toxstat version 3.5.

[CR46] Williams DA (1971). A test for differences between treatment means when several dose levels are compared with a zero dose control. Biometrics.

[CR47] Williams DA (1972). The comparison of several dose levels with a zero dose control. Biometrics.

