# Supplementary material for: Chronic Toxicity of Ferric Iron for North American Aquatic Organisms: Derivation of a Chronic Water Quality Criterion Using Single Species and Mesocosm Data
Source: Arch Environ Contam Toxicol. 2018 Jan 22;74(4):605–15. doi: 10.1007/s00244-018-0505-2 (PMC5893738; doi:10.1007/s00244-018-0505-2)
Supplement: Supplementary file 1 — Supplementary material 1 (DOCX 66 kb) [file 244_2018_505_MOESM1_ESM.docx]

**Chronic toxicity of ferric iron for North American aquatic organisms: derivation of a chronic water quality criterion using single species and mesocosm data**

Pete Cadmus^1^. Aquatic Toxicology Research Scientist.

Stephen F. Brinkman^1^. Aquatic Toxicology Research Scientist.

Melynda K. May^2^. Water Quality Program Coordinator.

1-Colorado Parks and Wildlife Aquatic Research Section. 317 West Prospect Rd. Fort Collins CO 80526 USA

2- Colorado Parks and Wildlife Water Resources Section. 6060 Broadway St. Denver, CO 80216 USA

Corresponding Author:

Pete Cadmus. Email: [pete.cadmus@state.co.us](mailto:pete.cadmus@state.co.us) Phone: US Code + 970-472-4332 Fax: 970-472-4457

**Contents:**

**Table S1: Water quality, survival and mass of Brown Trout and Mountain Whitefish**

**Table S2: Water quality, survival, development and mass of Boreal Toad Tadpoles**

**Table S3: Water quality, population growth and mass of *Lumbriculus***

**Table S4: Water quality, survival and mass of *Hexagenia***

**Table S5: Water quality, population growth and mass of Planarian worm.**

**Table S6: Water quality observations from mesocosm experiment**

**Table S7: No observed effect concentration (NOEC), lowest observed effect concentration (LOEC), maximum allowable toxicant concentration (MATC) and EC_20_ values for experiments used in the derivation of final chronic value**

**Table S8: Table of derivation equations and values for FCV excluding mesocosm results**

**Table S9: Table of derivation equations and values for FCV including mesocosm results**

**Supplemental Methods Narrative for derivation of FCV excluding mesocosm results**

Table S1. Water quality measurements and fry and egg survival for Brown Trout and Mountain Whitefish early-life stage Fe toxicity tests. Standard deviations are in parentheses. Asterisks denote treatment means significantly less than control (p<0.05). Fe reporting limits of <50 were reported for means of Fe between 0 and 49.

| Target Fe concentration (μg/L) | 0 | 625 | 1250 | 2500 | 5000 |
| --- | --- | --- | --- | --- | --- |
| Total Fe concentration (unfiltered) (μg/L) | <50  (13) | 658  (127) | 1329  (169) | 2438  (216) | 5146  (835) |
| Dissolved Fe concentration  (μg/L) | <50  (15) | <50  (34) | <50  (20) | <50  (18) | <50  (13) |
| Brown Trout hatch  (%) | 81.1  (7.9) | 64.4  (6.8) | 61.1  (10.3) | 65.6  (7.9) | 74.4  (6.8) |
| Brown Trout fry survival  (%) | 91.7  (6.8) | 100  (0) | 97.2  (3.9) | 94.4  (7.7) | 97.2  (3.9) |
| Brown Trout weight at termination  (g) | 0.295  (0.015) | 0.277  (0.014) | 0.271  (0.012) | 0.285  (0.011) | 0.248  (0.034) |
| Brown Trout biomass at termination (g) | 3.241 (0.080) | 3.327  (0.163) | 3.161 (0.251) | 3.229 (0.281) | 2.894 (0.310) |
| Mountain Whitefish  Hatch (%) | 86.7  (9.4) | 85.6  (1.6) | 90.0  (4.7) | 82.2  (4.2) | 88.9  (1.6) |
| Mountain Whitefish fry survival (%) | 84.4  (6.3) | 97.8  (3.1) | 86.7  (5.4) | 82.2  (12.6) | 66.7  (14.4) |
| Mountain Whitefish fry  weight (g) | 0.159 (0.010) | 0.141  (0.009) | 0.118*  (0.005) | 0.124 *  (0.010) | 0.099 *  (0.010) |
| Mountain Whitefish biomass at termination (g) | 2.005  (0.046) | 2.067  (0.141) | 1.532*  (0.076) | 1.544*  (0.368) | 0.980*  (0.206) |
| Alkalinity  (mg/L) | 33.8  (1.1) | 33.9  (0.8) | 33.6  (1.2) | 33.8  (1.1) | 33.5  (1.3) |
| pH  (SU) | 7.47  (0.09) | 7.50  (0.08) | 7.47  (0.07) | 7.49  (0.07) | 7.49  (0.08) |
| Incubation temperature  (°C) | 7.6  (0.2) | 7.5  (0.2) | 7.6  (0.2) | 7.6  (0.2) | 7.5  (0.2) |
| Post-hatch temperature  (°C) | 12.3 (0.1) | 12.4  (0.1) | 12.3 (0.1) | 12.3 (0.1) | 12.2 (0.1) |
| Conductivity  (μS/cm) | 74.8  (1.2) | 76.3  (1.4) | 79.1  (1.1) | 81.7  (2.4) | 90.8  (1.7) |
| Dissolved oxygen  (mg/L) | 9.50  (0.41) | 9.46  (0.50) | 9.46  (0.46) | 9.49  (0.47) | 9.48  (0.47) |

Table S2. Water quality measurements, survival, growth, development and mass of Boreal Toad tadpoles exposed to Fe for 35 days. Standard deviations are in parentheses. Asterisks denote treatment means significantly less than control (p<0.05). Fe reporting limits of <50 were reported for means of Fe between 0 and 49.

| Target Fe concentration (μg/L) | 0 | 500 | 1000 | 2000 | 4000 | 8000 |
| --- | --- | --- | --- | --- | --- | --- |
| Total Fe concentration (unfiltered) (μg/L) | <50  (13) | 654  (89) | 1073  (55) | 2044  (89) | 3831  (218) | 8115  (701) |
| Dissolved Fe concentration  (μg/L) | <50  (16) | <50  (41) | <50  (35) | <50  (21) | <50  (18) | <50  (13) |
| Survival  (%) | 100  (0) | 100  (0) | 95  (10) | 100  (0) | 60  (16)* | 35  (10)* |
| Mean length  (mm) | 56  (1) | 55  (2) | 55  (1) | 55  (1) | 52  (1) | 36  (2)* |
| Mean weight  (g) | 1.423 (0.052) | 1.490 (0.159) | 1.491 (0.100) | 1.493 (0.075) | 1.242 (0.100) | 0.414 (0.061)* |
| Gosner Stage | 39.4  (0.2) | 39.5  (0.2) | 39.7  (0.1) | 39.6 (0.5) | 39.0 (0.7) | 35.8 (0.3)* |
| Biomass  (g) | 7.113 (0.262) | 7.451 (0.794) | 7.058 (0.591) | 7.464 (0.377) | 3.737 (0.907)* | 0.728 (0.217)* |
| Alkalinity  (mg/L) | 34.9  (1.0) | 33.2  (0.6) | 33.9  (1.3) | 34.5  (1.9) | 35.4  (2.4) | 33.4  (1.3) |
| pH  (SU) | 7.10  (0.18) | 7.00  (0.07) | 7.15  (0.12) | 7.13  (0.15) | 7.13  (0.14) | 7.08  (0.14) |
| Conductivity  (μS/cm) | 109  (4) | 110  (4) | 113  (5) | 121  (5) | 131  (6) | 148  (14) |
| Dissolved oxygen  (mg/L) | 7.19  (0.34) | 7.49  (0.9) | 7.60  (0.45) | 7.36  (0.52) | 7.45  (0.45) | 7.65  (0.12) |

Table S3. Water chemistry measurements, population growth (from 15 individuals) and biomass of *Lumbriculus variegatus* after 35 days exposure to ferric iron. Standard deviations are in parentheses. Asterisks denote treatment means significantly less than control (p<0.05). Fe reporting limits of <100 were reported for means of Fe between 0 and 99.

| Target Fe concentration | 0 | 1000 | 2000 | 4000 | 8000 |
| --- | --- | --- | --- | --- | --- |
| Total Fe concentration (unfiltered) (μg/L) | <100  (13) | 593  (84) | 1145  (153) | 3087  (308) | 7592  (464) |
| Number of organisms | 113.0  (13.6) | 101.2  (29.1) | 80.8*  (29.4) | 51.4*  (14.4) | 25.6*  (6.2) |
| Biomass  (g) | 0.426  (0.062) | 0.463  (0.143) | 0.363  (0.126) | 0.186*  (0.062) | 0.094*  (0.029) |
| Hardness  (mg/L) | 44.6  (2.0) | 44.5  (1.7) | 44.1  (1.2) | 44.4  (1.1) | 44.2  (1.3) |
| Alkalinity  (mg/L) | 35.4  (2.2) | 34.6  (1.4) | 34.4  (1.3) | 34.6  (1.8) | 34.0  (2.6) |
| pH  (SU) | 7.86  (0.10) | 7.91  (0.11) | 7.88 (0.12) | 7.88  (0.13) | 7.85 (0.08) |
| Temperature  (°C) | 22.1  (0.1) | 22.1  (0.1) | 22.0  (0.3) | 22.2  (0.1) | 22.1  (0.1) |
| Conductivity  (μS/cm) | 114.0  (3.8) | 116.4  (5.8) | 121.2 (6.3) | 134.2 (4.8) | 165.7 (4.5) |
| Dissolved oxygen  (mg/L) | 7.55  (0.72) | 7.51  (0.78) | 7.57 (0.79) | 7.54 (0.77) | 7.62 (0.75) |

Table S4. Mean water chemistry measurements, survival and mass of *Hexagenia limbata* after 30 days exposure to ferric Fe. Standard deviations are in parentheses. No significant differences from the control group were observed. Fe reporting limits of <100 were reported for means of Fe between 0 and 99.

| Target Fe concentration | 0 | 500 | 1000 | 2000 | 4000 | 8000 |
| --- | --- | --- | --- | --- | --- | --- |
| Total Fe concentration (unfiltered) (μg/L) | <100  (38) | 464  (63) | 903  (100) | 1933  (171) | 3829  (195) | 7863  (244) |
| Survival  (%) | 86.1 (16.7) | 86.1 (16.7) | 83.3  (11.1) | 83.3 (11.1) | 91.7 (10.6) | 83.3 (11.1) |
| Weight  (mg) | 256 (18) | 219  (9) | 211  (42) | 254  (37) | 255  (49) | 224  (23) |
| Biomass  (mg) | 2050 (495) | 1705 (384) | 1600  (463) | 1921 (457) | 2124 (599) | 1680 (350) |
| Alkalinity  (mg/L) | 33.1  (1.2) | 33.2  (1.1) | 33.4  (1.1) | 33.0  (0.9) | 32.9  (0.7) | 33.4  (1.1) |
| pH  (SU) | 7.72  (0.04) | 7.77  (0.06) | 7.75  (0.07) | 7.76  (0.08) | 7.75  (0.04) | 7.76  (0.10) |
| Temperature  (°C) | 17.0  (0.6) | 17.0  (0.5) | 17.1  (0.6) | 17.0  (0.7) | 17.0  (0.6) | 17.0  (0.6) |
| Conductivity  (μS/cm) | 94.5  (9.5) | 97.0  (3.5) | 100.7  (4.1) | 103.8  (4.3) | 111.8  (7.7) | 133.8  (3.8) |
| Dissolved oxygen  (mg/L) | 7.61  (0.17) | 7.65  (0.17) | 7.58  (0.14) | 7.52  (0.18) | 7.62  (0.20) | 7.52  (0.17) |

Table S5. Mean water chemistry measurements, fissions, population growth and mass of *Dugesia dorotocephala* after 30 days exposure to ferric Fe. Standard deviations are in parentheses. No significant differences from the control group were observed. Fe reporting limits of <100 were reported for means of Fe between 0 and 99.

| Target Fe concentration | 0 | 5000 | 1000 | 20000 | 30000 | 40000 |
| --- | --- | --- | --- | --- | --- | --- |
| Total Fe concentration (unfiltered) (μg/L) | <100  (38) | 2502  (58) | 5050  (183) | 10214  (351) | 20566  (666) | 40134  (1106) |
| Population growth  (%) | 145.83  (25.00) | 141.67  (21.52) | 108.33  (16.67) | 162.50  (34.36) | 150.00  (13.61) | 137.50  (15.96) |
| Fissions | 2.75  (1.5) | 2.50  (1.29) | 0.50  (1.00) | 3.75  (2.06) | 3.00  (0.82) | 2.25  (0.96) |
| Biomass  (mg) | 15.65  (0.72) | 15.78  (2.29) | 15.45  (1.51) | 16.45  (1.88) | 13.60  (1.88) | 14.98  (1.18) |
| Alkalinity  (mg/L) | 75.13  (8.71) | 76.80  (14.67) | 75.33  (6.33) | 74.07  (5.55) | 74.07  (3.75) | 72.60  (2.16) |
| pH  (SU) | 7.19  (0.14) | 7.25  (0.17) | 7.22  (0.07) | 7.27  (0.05) | 7.26  (0.15) | 7.26  (0.14) |
| Temperature  (°C) | 22.63  (1.80) | 22.67  (1.89) | 22.80  (1.93) | 22.80  (2.10) | 22.77  (2.25) | 22.97  (2.25) |
| Conductivity  (μS/cm) | 398.67  (44.60) | 454.67  (59.34) | 466.33  (37.58) | 512.33  (40.53) | 565.00  (48.12) | 620.67  (47.25) |
| Dissolved oxygen  (mg/L) | 4.18  (3.05) | 4.34  (2.82) | 4.66  (2.63) | 4.61  (2.61) | 4.60  (2.64) | 4.65  (2.57) |

Table S6. Mean water chemistry measurements from mesocosm experiment exposing naturally colonized communities of benthic macroinvertebrates to ferric Fe for 10 days. * = below instrument detection limits.

| Target Iron concentration | 0 | 400 | 1000 | 2500 | 6250 | 15000 |
| --- | --- | --- | --- | --- | --- | --- |
| Total Fe concentration (unfiltered) (μg/L) | 124 *  (6) | 446  (39) | 944  (73) | 2425  (83) | 5238  (354) | 14073  (450) |
| Hardness  (mg/L) | 35  (0.6) | 35.7  (0.7) | 32.7  (0.9) | 32.7  (2.8) | 33.75  (1.0) | 34.33  (1.2) |
| Alkalinity  (mg/L) | 27.25  (2) | 29  (0.5) | 28  (1.5) | 32.6  (2.9) | 25.7  (1.2) | 24.2  (1.6) |
| pH  (SU) | 7.24  (0.10) | 7.22  (0.09) | 7.27  (0.10) | 7.23  (0.10) | 7.25  (0.12) | 7.23  (0.09) |
| Temperature  (°C) | 11.71  (0.26) | 11.75  (0.28) | 11.63  (0.26) | 11.75  (0.26) | 11.92  (0.33) | 11.76  (0.28) |
| Conductivity  (μS/cm) | 90.4  (9) | 95.4  (9) | 100.0  (9) | 101.0  (10) | 128.1  (9) | 204.8  (11) |
| Dissolved Oxygen  (mg/L) | 7.7  (0.1) | 7.5  (0.1) | 7.6  (0.1) | 7.5  (0.1) | 7.2  (0.1) | 7.2  (0.1) |

Table S7: No observed effect concentration (NOEC), lowest observed effect concentration (LOEC), maximum allowable toxicant concentration (MATC) and EC_20_ values. Underlined values used as chronic value for derivation of Final Chronic Value. Fe concentrations in µg/L total or total recoverable Fe.

| Scientific name | Common name | NOEC | LOEC | MATC | EC_20_ | Reference |
| --- | --- | --- | --- | --- | --- | --- |
| *Dugesia dorotocephala* | Planarian | >40134 |  | >40134 | >40134 | This report |
| *Orconectes limosus* | Crayfish |  |  | 22000^c^ | ^b^ | Boutet and Chaisemartin 1973 |
| *Chironomus riparius* | Midge | 15000 | 30000 | 21213 | 19818 | Radford 1997 |
| *Salvelinus fontinalis* | Brook trout | 7800 | 13420 | 10231 | 9237 | Sykora et al. 1975 |
| *Hexagenia limbata* | Mayfly | >7863 |  | >7863 | >7863 | This report |
| *Salmo trutta* | Brown trout | >5146 |  | >5146 | >5146 | This report |
| *Oncorhynchus kisutch* | Coho salmon | 2830 | 4635 | 3621 | 4870 | Smith and Sykora 1976 |
| *Oncorhynchus kisutch* | Coho salmon | >3300 |  | >3300 | >3300 | Brenner and Cooper 1978 |
| *Oncorhynchus kisutch* | Coho salmon | 2000 | 4250 | 2915 | ^b^ | Updegraff and Sykora 1976 |
| *Oncorhynchus mykiss* | Rainbow trout | 1000 | 2200 | 1483 | ^b^ | Goettl and Davies 1977 |
| *Oncorhynchus mykiss* | Rainbow trout | >7500 |  | >7500 | >7500 | Steffens et al. 1993 |
| *Bufo boreas* | Boreal toad | 2044 | 3831 | 2798 | 3145 | This report |
| *Daphnia magna* | Cladoceran |  |  | 4380^d^ | ^b^ | Biesinger and Christensen 1972 |
| *Daphnia pulex* | Cladoceran | 700 | 1310 | 958 | 979^a^ | Birge et al. 1985 |
| *Prosopium williamsoni* | Mountain whitefish | 658 | 1329 | 935 | 1318 | This report |
| *Lumbriculus variegates* | Worm | 593 | 1145 | 880 | 870 | This report |
| *Pimephales promelas* | Fathead minnow | 316 | 1008 | 569 | 910 | Birge et al. 1985 |
| *Pimephales promelas* | Fathead minnow |  | <2000 | <2000 | 520 | Smith et al. 1973 |

^a^ Insufficient partial effects for reliable estimate of EC_20_. Value reported for comparison purpose only.

^b^ Insufficient data reported to run TRAP for EC_20_ value.

^c^ 30d LC50 reported by authors used as MATC.

^d^ 21d EC16 reported by authors used as MATC.

Table S8: Derivation of Final Chronic Value per Stephen et al. (EPA 1985) using single species experiments including spreadsheet equations in Microsoft Excel format.

N= Number of Genera = 12

R= Rank

| RANK | GENUS | GMCV | | Ln(GMCV) | Ln(GMCV)**2 | P=R/N+1 | P**0.5 |
| --- | --- | --- | --- | --- | --- | --- | --- |
| 4 | *Daphnia* | 2048 | | 7.6246 | 58.1348 | 0.3077 | 0.5547 |
| 3 | *Prosopium* | 1318 | | 7.1839 | 51.6080 | 0.2308 | 0.4804 |
| 2 | *Lumbriculus* | 870 | | 6.7685 | 45.8125 | 0.1538 | 0.3922 |
| 1 | *Pimephales* | 688 | | 6.5338 | 42.6904 | 0.0769 | 0.2774 |
|  |  |  | |  |  |  |  |
|  | SUM | | | 28.1108 | 198.2457 | 0.7692 | 1.7047 |
|  | SUM SQUARED | | | 790.2155 | 39301.3616 | 0.5917 | 2.9059 |
|  |  |  | |  |  |  |  |
|  |  |  | |  |  |  |  |
| S2 = SUM(LnGMCV)2 - (SUMLnGMCV)2/4/SUM(P)-SUM(P**0.5)**2/4 | | | | | 16.1802 |  | 4.022 |
|  |  |  |  | |  |  |  |
| L=(SUM(LnGMCV)-S*SUM(P**0.5))/4 | | |  | | 5.3135 |  |  |
|  |  |  |  | |  |  |  |
| A=S*SQRT(0.5) +L | |  |  | | 6.2129 |  |  |
|  |  |  |  | |  |  |  |
| FCV=EXP(A) | |  |  | | 499 |  |  |

Literature Cited:

Stephan CE, Mount DI, Hansen DJ, Gentile JR, Chapman GA, Brungs WA (1985) Guidelines for deriving numerical standards for the protection of aquatic organisms and their uses. PB85-227049. USEPA, Springfield, VA

Table S9: Derivation of Final Chronic Value per Stephen et al. (EPA 1985) using single species experiments including spreadsheet equations in Microsoft Excel format.

N= Number of Genera = 26

R= Rank

| RANK | GENUS | | GMCV | Ln(GMCV) | Ln(GMCV)**2 | P=R/N+1 | P**0.5 |
| --- | --- | --- | --- | --- | --- | --- | --- |
| 4 | *Pimephales* | | 688 | 6.5338 | 42.6904 | 0.1481 | 0.3849 |
| 3 | *Microsema sp.* | | 356.29 | 5.8757 | 34.5244 | 0.1111 | 0.3333 |
| 2 | *Epeorus sp.* | | 334.5 | 5.8126 | 33.7867 | 0.0741 | 0.2722 |
| 1 | Tanytarsini (Tribe) | | 233.65 | 5.4538 | 29.7442 | 0.0370 | 0.1925 |
|  |  | |  |  |  |  |  |
| SUM |  | |  | 23.6760 | 140.7457 | 0.3704 | 1.1828 |
| SUM SQUARED | | |  | 560.5527 | 19809.3584 | 0.1372 | 1.3991 |
|  |  | |  |  |  |  |  |
|  |  | |  |  |  |  |  |
| S2 = SUM(LnGMCV)2 - (SUMLnGMCV)2/4/SUM(P)-SUM(P**0.5)**2/4 | | | | | 29.5102 |  | 5.432 |
|  |  |  | |  |  |  |  |
| L=(SUM(LnGMCV)-S*SUM(P**0.5))/4 | | | |  | 4.3126 |  |  |
|  |  |  | |  |  |  |  |
| A=S*SQRT(0.5) +L | |  | |  | 5.5273 |  |  |
|  |  |  | |  |  |  |  |
| FCV=EXP(A) | |  | |  | 251 |  |  |

Literature Cited:

Stephan CE, Mount DI, Hansen DJ, Gentile JR, Chapman GA, Brungs WA (1985) Guidelines for deriving numerical standards for the protection of aquatic organisms and their uses. PB85-227049. USEPA, Springfield, VA

Supplemental Information Methods Narrative

Chronic Iron Toxicity Data for Derivation of Final Chronic Value using Single Species Trials

Chronic iron toxicity tests were identified using United States Environmental Protection Agency’s Ecotox database (USEPA 2015a) and other electronic literature databases and then screened using the following criteria: 1. Species of test organism used must exist in freshwater systems in North America, 2. The duration of the test was sufficiently long to detect sublethal effects (≥25 days or ≥7days for Daphnids), 3. Ferrous iron was used as the toxicant. This third criterion was selected because ferrous iron and its precipitates are the overwhelming predominant form of iron in circumneutral oxygenated waters, 4. Toxicity tests were conducted at pH between 6.5 and 9.0 in order to minimize confounding effects of pH on toxicity results (see e.g. Radford 1997).

Toxicity tests meeting these criteria were relatively few but when added to tests reported here met the minimum of eight families needed to derive a chronic criterion for protection of aquatic life (Stephan et al. 1985). Test organisms included Salmonidae (*Oncorynchus kisutch, Oncorynchus mykiss, Prosopium williamsoni, Salvelinus fontinalis, Salmo trutta*), another fish family in class Osteichthyes (*Pimephales promelas*), a third family in Chordata (*Bufo boreas*), planktonic crustaceans (*Daphnia magna, Daphnia pulex*), benthic crustaceans (*Orconectes limosus*), Arthropoda (*Chironomus riparius, Hexagenia limbata*), a family in a phylum other than Arthropoda or Chordata (*Lumbriculus variegatus*), and finally a family in any order of insect or phylum not already represented (*Dugesia dorotocephala*; Supplemental Table 1). A study on the avoidance of *Oncorynchus kisutch* to ferric iron suspensions (Updegraff and Sykora 1976) did not meet the criterion for test duration but was included because avoidance was considered to be a relevant chronic endpoint.

Most studies reported maximum allowable toxicant concentrations (MATC), calculated as the geometric mean of a no observed effect concentration (NOEC) and lowest observed effect concentration (LOEC) (Table 6). Many studies reported data in sufficient detail to utilize USEPA’s Toxicity Relationship Analysis Program version 1.30a (TRAP; USEPA 2015b) to perform regression analyses of results. Log transformed threshold sigmoid was routinely a good fit for data from single species trials. In such instances, iron concentrations estimated to cause a 20 percent reduction in response relative to control treatments (EC_20_) were calculated to be consistent with current USEPA procedures for development of chronic criteria. EC_20_s were generally in good agreement with MATCs and were always between NOECs and LOECs reported by the authors (Table 6).

Five iron toxicity tests did not detect an adverse effect at the highest iron concentration tested. In these instances, an EC_20_ or MATC could not be determined. If all of the iron exposure concentrations were very low, unbounded “greater than” toxicity thresholds would provide little information. However, if the highest exposure concentration was high, the “greater than” toxicity value indicates a species with high resistance to iron. Stephan et al. (1985) noted that unbounded “greater than” acute toxicity values should be used because excluding results from such resistant species would unnecessarily lower the Final Acute Value. This reasoning is equally applicable to calculating a Final Chronic Value. The five unbounded “greater than” toxicity values were deemed sufficiently high to indicate resistant species and were used as the chronic value for *Oncorhynchus mykiss* (>7500 µg/L; Steffens et al.1993), *Oncorhynchus kisutch* (>3300 µg/L; Brenner and Cooper 1978), *Salmo trutta* (>5146 µg/L)*, Hexagenia limbata* (>7863 µg/L) and *Dugesia dorotocephala* (>40,126 µg/L).

Chronic values used to calculate the Final Chronic Value (FCV) were based on EC_20_ values in instances where reliable estimates could be made. Otherwise, chronic values were MATCs or other toxicity threshold values reported by the authors (21d EC16 for *Daphnia magna;* Biesinger and Christensen 1972 and 30d LC_50_ for *Orconectes limosus*; Boutet and Chaisemartin 1973).

Efforts were made to include data from as many experiments as possible given the limited number of relevant studies. After reviewing the toxicity reports, results of three studies were excluded from derivation of the Final Chronic Value (FCV). Dave (1984) reported a maximum allowable toxicant concentration (MATC) of 181 µg/L for *Daphnia magna* which was much lower than other chronic values and deemed inconsistent with other chronic values reported for Daphnids. Unpublished toxicity results for *Chematopsyche* and *Gammarus minus* (Sykora 1972) lacked a clear concentration-response and were also excluded.

Two other Daphnid studies were excluded because we could find no record of their presence in North America. A MATC for *Daphnia carinata* was 2419 µg/L (Van Dam et al. 1998). Randall et al. (1999) report a “safe limit” of particulate iron of 1690 µg/L for *Daphnia longispina*. While the MATCs for these Daphnids were excluded, it is worth noting that they fell within the range of 958 µg/L for *Daphnia pulex* and 4380 µg/L for *Daphnia magna*.

**Literature Cited**

Boutet C, Chasemartin C (1973) Proprietes toxiques specifiques des sels metalliques chez Austropotamobius pallipes pallipes et Orconectes limosus. Comptes Rendus des Seances de la Societe de Biologie et de ses Filiales 167:1933-1938

Dave G (1984) Effects of waterborne iron on growth, reproduction, survival and haemoglobin in Daphnia magna. Comp Biochem Phys 78C:433-438.

Radford NP (1997) Ecotoxicological impact of iron III sulphate dosing on chironomid cultures and profundal reservoir communities. Doctoral Thesis, University of Leicester

Randall S, Harper D, Brierley B (1999) Ecological and ecophysiological impacts of ferric dosing in reservoirs. Hydrobiologia 395/396: 355-364

Stephan CE, Mount DI, Hansen DJ, Gentile JR, Chapman GA, Brungs WA (1985) Guidelines for deriving numerical standards for the protection of aquatic organisms and their uses. PB85-227049. USEPA, Springfield, VA

Sykora JL, Smith EJ, Synak M, Shapiro MA (1975) Some observations on spawning brook trout (Salvalinus fontinalis Mitchell) in lime neutralized iron hydroxide suspensions. Water Res 9:451-458

Updegraff KF, Sykora JL (1976) Avoidance of lime-neutralized iron hydroxide solutions by coho salmon in the laboratory. Environ Sci Technol 10:51-54

USEPA (2015a) ECOTOX User Guide: ECOTOXicology Knowledgebase System. Version 4.0. https://cfpub.epa.gov/ecotox/blackbox/help/userhelp4.pdf. Accessed 28 April 2017

USEPA (2015b) Toxicity Relationship Analysis Program (TRAP Version 1.30a). USEPA. Duluth, MN

Van Dam RA, Barry MJ, Ahokas JT, Holdway DA (1998). Effects of water-borne iron and calcium on the toxicity of diethylenetriamine pentaacetic (DTPA) to Daphnia carinata. Aquat. Toxicol. 42:49-66
